# Morphological and immunohistochemical identification of epithelial-to-mesenchymal transition in clinical prostate cancer

**DOI:** 10.18632/oncotarget.4177

**Published:** 2015-05-19

**Authors:** Kimberley Kolijn, Esther I. Verhoef, Geert J.L.H. van Leenders

**Affiliations:** ^1^ Department of Pathology, Erasmus Medical Centre, Rotterdam, The Netherlands

**Keywords:** epithelial-to-mesenchymal transition, ill-defined, N-cadherin, prostate cancer

## Abstract

Epithelial-to-mesenchymal transition (EMT) is a process known to be associated with aggressive tumor behavior, metastasis and treatment resistance. It is characterized by coincidental upregulation of mesenchymal markers such as vimentin, fibronectin and N-cadherin concurrent with E-cadherin downregulation. Studies on EMT are generally performed in cell lines and mouse models, while the histopathological and phenotypical properties in clinical prostate cancer (PCa) are still unclear.

The objective of this study was to identify EMT in PCa patients. We demonstrated that N-cadherin, vimentin and fibronectin were generally not co-expressed in corresponding tumor regions. Immunofluorescent double stainings confirmed that co-expression of mesenchymal markers was uncommon, as we found no prostate cancer cells that co-expressed N-cadherin with fibronectin and only rare (<1%) cells that co-expressed N-cadherin with vimentin. Downregulation of E-cadherin was demonstrated in all N-cadherin positive tumor cells, but not in vimentin or fibronectin positive tumor cells. We further analyzed N-cadherin expression in morphologically distinct PCa growth patterns in a radical prostatectomy cohort (*n* = 77) and found that N-cadherin is preferentially expressed in ill-defined Gleason grade 4 PCa. In conclusion, we demonstrate that N-cadherin is the most reliable marker for EMT in clinical PCa and is preferentially expressed in ill-defined Gleason grade 4 growth pattern.

## INTRODUCTION

**Figure 1:**
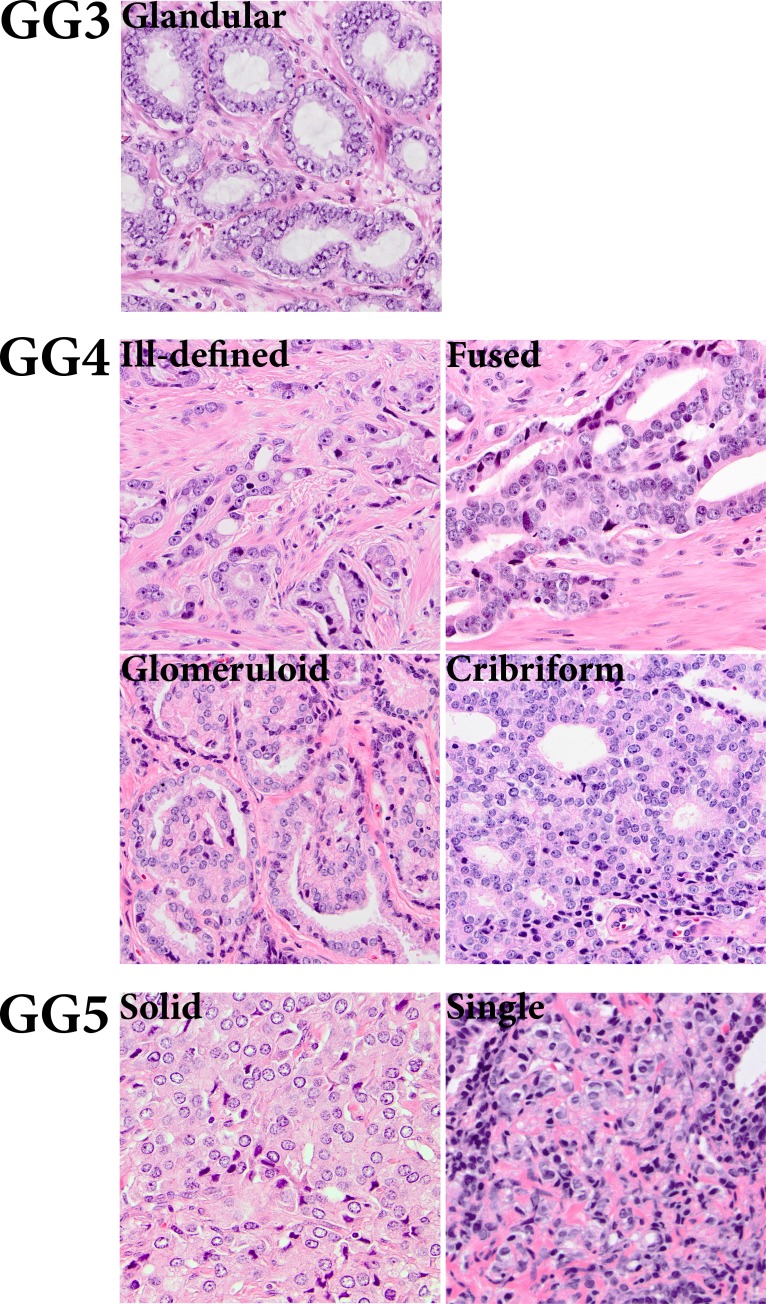
Gleason grade growth patterns of clinical prostate cancer Gleason grade 3 (GG3) consists of well-delineated round to ellipsoid tumor glands. Gleason grade 4 (GG4) comprises the following growth patterns: ill-defined, fused, glomeruloid and cribriform. Gleason grade 5 (GG5) shows a solid tumor growth pattern or growth in single cells and cords (20x, original magnification).

The grade of differentiation in clinical PCa according to the modified Gleason scoring system is one of the most important predictive factors for disease behavior and therapeutic decision-making [1-3]. The Gleason grading system is solely based on architectural tumor growth patterns. While Gleason grade 1-3 PCa is composed of well-delineated malignant glands, various growth patterns classify as Gleason grade 4, such as ill-defined, fused, glomeruloid and cribriform (Figure 1) [1-3]. Despite the widespread application of Gleason grading in clinical practice, little is known on the molecular background of specific Gleason grade growth patterns.

Understanding the molecular-biological processes by which tumor cells invade surrounding tissues and metastasize to distant sites, is an ongoing challenge in cancer research, as metastasis is the major cause of death for cancer patients. Metastasis requires cells to loose cellular connections and become migratory, which can be driven by the process of EMT [4]. Expression of E-cadherin (cadherin-1) is the keystone of epithelial cell state and is downregulated during EMT [4]. In many carcinomas including PCa, EMT is associated with invasive and aggressive behavior, metastasis to distant locations and treatment resistance [5-11]. Especially the shift from E-cadherin to N-cadherin (cadherin-2) expression correlates with cancer progression and metastasis [12-17].

While EMT in PCa has mainly been studied in cell line models, the morphological and phenotypic substrate of EMT in PCa patients is still unclear. In colon carcinoma, cancer cells undergoing EMT are characterized by spindle-shaped morphology similar to *in vitro* studies [18]. In conventional clinical PCa, however, spindle-shaped tumor cells are not observed and thus the morphological substrate of EMT is still unclear in these tumors. The aim of this study was to investigate the morphology and location of EMT in clinical PCa. We demonstrate for the first time that EMT in PCa is characterized by N-cadherin expression in morphologically ill-defined Gleason grade 4 PCa glands.

## RESULTS

### EMT marker expression in benign and malignant prostate epithelium

While EMT in cell culture models is characterized by simultaneous up- and downregulation of markers such as vimentin, N-cadherin and E-cadherin, it is not known whether synchronous expression also occurs within cell populations undergoing EMT in clinical PCa. First, we studied the expression of EMT markers vimentin, fibronectin, N-cadherin and E-cadherin in a set of 23 radical prostatectomy (RP) specimens to determine which marker is most representative for EMT in clinical specimens and whether EMT markers were co-expressed within the same tumor areas. The clinicopathologic features of the cohort (primary set) are shown in Table 1.

**Table 1:** Clinicopathologic features of the primary and extended RP cohorts

		Mean (Median; range) or n (%)	
Parameter		Primary set (n=23)	Extended set (n=77)
Age		66 (67; 63-69)	66 (67; 51-79)
PSA level (ng/mL)		22 (15; 12-32)	17 (10; 2-97)
Gleason score	5	0 (0%)	1 (1%)
	6	2 (9%)	12 (16%)
	7 (3+4)	8 (34%)	33 (43%)
	7 (4+3)	3 (13%)	14 (18%)
	8	5 (22%)	9 (12%)
	9	5 (22%)	8 (10%)
pT stage (WHO 2009)	pT2	0 (0%)	25 (33%)
	pT3a	18 (78%)	41 (53%)
	pT3b	5 (22%)	11 (14%)
	pT4	0 (0%)	0 (0%)
Surgical margin	Negative	13 (57%)	49 (64%)
	Positive	10 (43%)	28 (36%)

PSA = prostate specific antigen

Cytoplasmic vimentin was abundantly, although not homogeneously, expressed in normal fibromuscular prostate stroma of all patients, as well as in sporadic benign luminal epithelial cells of patients (22/23, 96%) (Table 2). In benign epithelium, only one case (1/23, 4%) demonstrated complete absence of vimentin expression. Vimentin expression in benign epithelium was positive in < 1% of luminal cells in the majority of patients (18/23, 78%), while expression in 1-4% or > 4%-10% of luminal cells occurred in 2/23 (9%) cases each. Vimentin expression was observed in 9/23 (39%) PCa specimens (Table 2); 7 tumors showed expression in < 1% of malignant cells (30%) and 2 cases in between 1% and 4% of malignant cells (9%). In most tumors (14/23; 61%), however, no vimentin expression was observed. The percentage of vimentin positive cells in benign epithelium was significantly higher than in PCa (*p* < 0.001).

**Table 2:** Immunohistochemical expression of vimentin, fibronectin and N-cadherin in benign and malignant prostate epithelial cells in the primary RP cohort

Marker	Epithelium type	Percentage of positive epithelial cells; n (%)			
		0	<1%	1 - 4%	>4% - 10%
Vimentin	Benign (n=23)	1 (4%)	18 (78%)	2 (9%)	2 (9%)
	PCa (n=23)	14 (61%)	7 (30%)	2 (9%)	0 (0%)
Fibronectin	Benign (n=23)	1 (4%)	22 (96%)	0 (0%)	0 (0%)
	PCa (n=23)	2 (9%)	19 (82%)	0 (0%)	2 (9%)
N-cadherin	Benign (n=23)	11 (48%)	12 (52%)	0 (0%)	0 (0%)
	PCa (n=23)	10 (44%)	9 (39%)	1 (4%)	3 (13%)

PCa = prostate cancer

Fibronectin was abundantly expressed in the cytoplasm of prostate stromal cells and in < 1% of benign luminal epithelial cells (22/23, 96%) (Table 2). In PCa tissues, expression of fibronectin occurred most frequently in < 1% of tumor cells (19/23, 82%), while 2 cases were entirely negative (2/23, 9%) or showed expression in > 4%-10% of PCa cells (2/23; 9%) (Table 2). Expression of fibronectin was not statistically different in benign and malignant prostate epithelium (*p* = 0.28).

N-cadherin was expressed in all nerve fibers, which served as positive internal control in all cases. Expression in < 1% of benign luminal epithelial cells was present in 12/23 samples (52%), while the remaining cases were entirely negative (11/23, 48%) (Table 2). In PCa, N-cadherin expression was absent in 10 cases (44%) or present in < 1% (9/23, 39%), 1%-4% (1/23, 4%) or > 4%-10% (3/23, 13%) of PCa cells (Table 2). Expression of N-cadherin was statistically similar in benign and malignant prostate epithelium (*p* = 0.21), although high expression ( > 1-10%) was exclusive to malignant epithelium. None of the EMT markers showed expression in more than 10% of benign or malignant epithelial cells.

**Figure 2:**
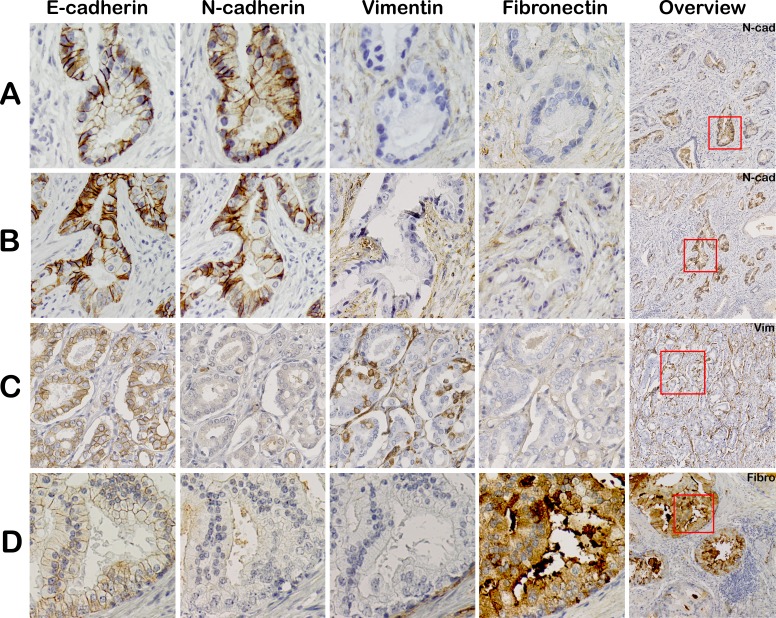
Mesenchymal EMT markers are not expressed in corresponding regions in clinical PCa Consecutive slides were stained for EMT markers: E-cadherin, N-cadherin, vimentin and fibronectin. Tumor areas positive for N-cadherin were negative for vimentin and fibronectin (panel **A** and **B**). Vimentin (panel **C**) and fibronectin (panel **D**) positive tumor areas also did not co-express any of the other EMT markers (20x, original magnification). Overview slides demonstrate Gleason score 3+4 = 7 (ill-defined; panel **A**, **B**), 3+4=7 (fused; panel **C**) and 4+3 = 7 (cribriform; panel **D**).

Comparison of vimentin, fibronectin and N-cadherin expression patterns in consecutive PCa sections did not reveal overlap of staining within corresponding areas (Figure 2). A hallmark event of EMT is downregulation of E-cadherin in epithelial cells. Areas with a complete loss of E-cadherin were not identified immunohistochemically in any case (0/23), although focal variation in expression intensity was observed.

### Immunofluorescent co-expression analysis of EMT markers

**Figure 3:**
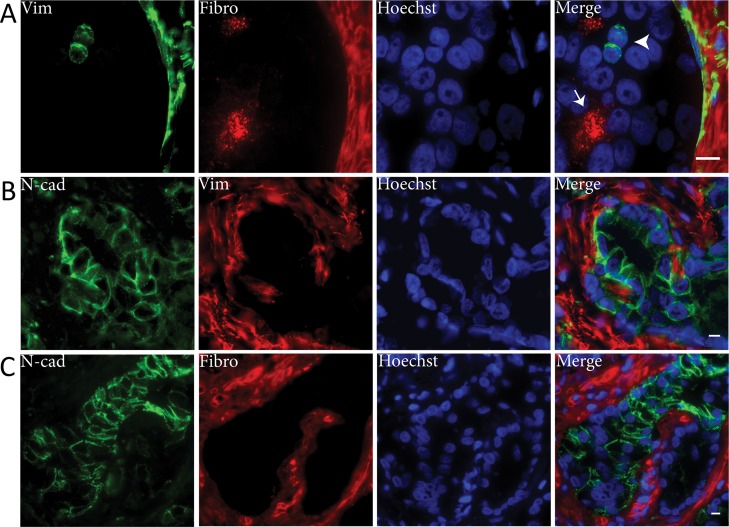
Mesenchymal EMT markers are not co-expressed in clinical PCa Immunofluorescent double stainings demonstrated that vimentin (Vim) and fibronectin (Fibro) were not co-expressed (panel A). Vimentin positive PCa cells were fibronectin negative (arrowhead) and vice versa (arrow). N-cadherin (N-cad) was neither co-expressed with vimentin (panel B) or fibronectin (panel C). Original magnifications 100x (**A**) and 63x (**B**, **C**). Scale bar = 10 μm.

To verify that EMT markers vimentin, fibronectin and N-cadherin were not expressed within the same cell population, we performed immunofluorescent co-expression analysis in PCa samples (Figure 3). Vimentin did not co-localize with fibronectin (Figure 3A). In only one patient, < 1% of N-cadherin positive PCa cells were also positive for vimentin, while the vast majority of N-cadherin positive cells were vimentin negative (Figure 3B). Co-localization of fibronectin and N-cadherin was not observed (Figure 3C). Therefore, vimentin, fibronectin and N-cadherin were generally expressed in independent cell populations.

**Figure 4:**
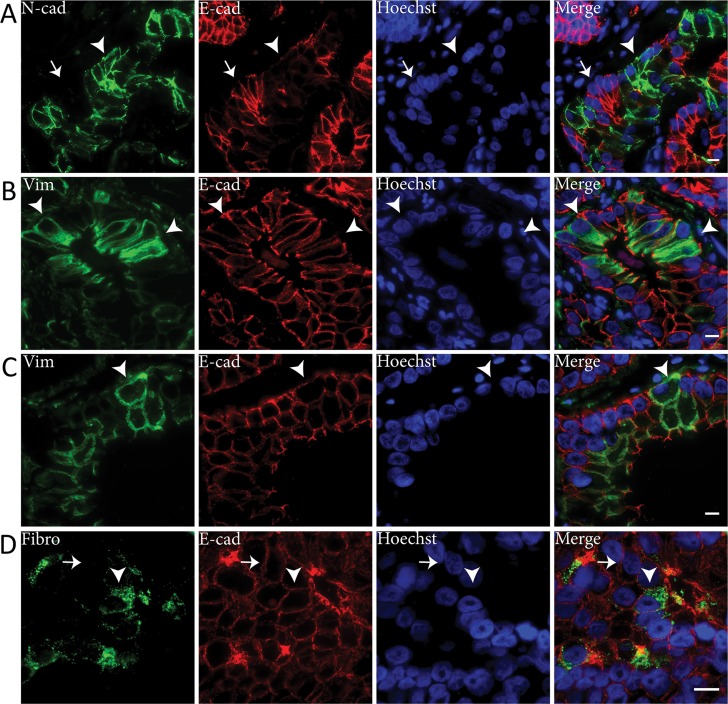
E-cadherin is downregulated in N-cadherin positive tumor cell clusters in clinical PCa Immunofluorescent double stainings showed E-cadherin (E-cad) downregulation in N-cadherin (N-cad) positive cell clusters (arrowhead) (panel A). N-cadherin negative cells demonstrated normal E-cadherin levels at the membrane (arrow). Vimentin (Vim) positive PCa cells generally expressed normal levels of E-cadherin (arrowheads) (panel B), although E-cadherin was downregulated in some vimentin positive cells (arrowheads) (panel C). Fibronectin (Fibro) positive PCa cells expressed similar E-cadherin levels (arrowhead) as fibronectin negative PCa cells (arrow) (panel D). Original magnifications 63x (**A**-**C**) and 100x (**D**). Scale bar = 10 μm.

To determine which of the markers was most representative for EMT in clinical specimens, we performed immunofluorescent co-expression analysis with E-cadherin (Figure 4). Downregulation of E-cadherin was seen in all malignant cell clusters that expressed N-cadherin, indicative of cadherin switching (Figure 4A) [15, 19]. E-cadherin expression was high in the majority of vimentin positive PCa cells (Figure 4B). In a subpopulation (circa 10%) of vimentin positive tumor cells, however, E-cadherin was slightly decreased (Figure 4C). None of the fibronectin positive PCa cells showed E-cadherin downregulation (Figure 4D). Since expression of N-cadherin coincided with vast E-cadherin downregulation, N-cadherin was considered the most reliable mesenchymal marker for EMT in clinical PCa.

### N-cadherin expression in clinical PCa

EMT is preferentially occurring at the invasive front of various human cancers, such as oral squamous cell carcinoma and colorectal adenocarcinoma [20, 21]. In these tumor types, EMT is characterized by a spindle-like morphology of tumor cells and gain of mesenchymal markers. Spindle-shaped tumor cells, however, are not pathologically recognized in conventional PCa. To determine whether EMT as identified by N-cadherin expression was also related to a specific growth pattern in clinical PCa, we quantified its expression in distinctive PCa growth patterns, as defined by the WHO 2009, in a set of 77 RP specimens (Figure 1) [1-3]. Expression of N-cadherin occurred significantly more frequent in ill-defined (27/70, 39%) than in fused (3/50, 6%; *p* < 0.001) or cribriform (2/29, 7%; *p* = 0.001) Gleason grade 4 PCa (Table 3). Although N-cadherin expression occurred more often in ill-defined PCa (27/70, 39%) than in glandular Gleason grade 3 (21/75, 28%; *p* = 0.22), glomeruloid Gleason grade 4 (2/10, 20%; *p* = 0.31), solid fields (0/4, 0%; *p* = 0.29), cords and single cells (2/8, 25%; *p* = 0.70) Gleason grade 5 PCa, the difference did not reach statistical significance. However, the overall percentage of N-cadherin positive cells was significantly higher in ill-defined glands (median 10%; range 1-100%) than in Gleason grade 3 (median 1%; range 1-5%; *p* < 0.001) PCa. Due to the low number of N-cadherin positive fused (*n* = 3), glomeruloid (*n* = 2), solid fields (*n* = 0), cords and single cells (*n* = 2) PCa growth patterns, statistical analysis was not performed in these cases. Expression of N-cadherin was neither confined to the tumor boundary (invasive front) nor enriched in extra-prostatic (pT3) disease (*p* = 0.16).

**Table 3:** Expression of N-cadherin in distinct PCa growth patterns in the extended RP cohort

Growth pattern	Frequency of growth pattern	Presence of N-cadherin expression	Median (mean; range) N-cadherin expression in cases
**Gleason grade 3**			
Glandular	75 (97%)	21/75 (28%)	1% (1%; 1-5%)
**Gleason grade 4**			
Ill- defined	70 (91%)	27/70 (39%)	10% (23%; 1-100%)
Fused	50 (65%)	3/50 (6%)	5% (7%; 5-10%)
Cribriform	29 (28%)	2/29 (7%)	6% (6%; 1-10%)
Glomeruloid	10 (13%)	2/10 (20%)	2% (2%; 1-2%)
**Gleason grade 5**			
Cords, single cells	8 (10%)	2/8 (25%)	5% (5%; 5%)
Solid fields	4 (5%)	0/4 (0%)	0%

## DISCUSSION

EMT is an important biological process in embryogenesis, tissue homeostasis and tumor progression. It has been extensively studied in clinical colorectal cancer, where it is recognized as spindle-shaped tumor cells within desmoplastic stroma at the invasive front [18, 22-24]. Studies on EMT in human prostate tissues generally encompass immunohistochemical analysis of single markers such as E-cadherin, N-cadherin, β-catenin, fibronectin, Slug, Snail, Twist, vimentin, ZEB1 and ZEB2 in relation to clinicopathologic features including death of disease and metastasis [12, 19, 25-30].

Various groups have pointed at the importance of EMT and N-cadherin expression for the progression of clinical PCa [7, 19, 25, 26, 31, 32]. Expression of N-cadherin has been found in 0% up to 54% of PCa patients [19, 26, 27]. We found that 48% of PCa expressed N-cadherin, which is in line with other reports. Similar to previous studies, we have demonstrated that E-cadherin, a key EMT marker, was downregulated in N-cadherin positive tumor cells, which is often referred to as cadherin switching [12, 19]. In this study, we found that EMT as identified by N-cadherin expression occurred preferentially in ill-defined PCa glands. Ill-defined PCa glands correspond to tumor glands with irregular outline and are considered a subtype of modified Gleason grade 4 PCa [1]. While we also found expression in glandular Gleason 3, the median percentage of positive N-cadherin cells was significantly lower as compared to ill-defined glands. As ill-defined modified Gleason grade 4 PCa glands have an irregular contour and are strongly associated with immunohistochemical cadherin switching, they putatively represent the morphological substrate of EMT in clinical PCa.

Although N-cadherin overexpression occurred preferentially in Gleason grade 4 ill-defined growth pattern, a significant number of ill-defined structures did not express N-cadherin. It is possible that other cadherins such as OB-cadherin (cadherin-11) are upregulated in these prospective EMT regions [33, 34]. EMT has also been implicated to play a role in therapy resistance [6, 35, 36]. Expression of mesenchymal markers including vimentin and fibronectin was increased and E-cadherin was decreased upon androgen deprivation in PCa patients [6, 35]. Antibodies directing against N-cadherin inhibited tumor growth, metastasis and castration resistance of PCa cells in xenografted mice [36]. Since the stromal and inflammatory background in lymph node and bone metastasis is different from prostate stroma, in this study we specifically set out to investigate EMT in the context of its naive prostate background.

Although N-cadherin, vimentin and fibronectin are generally all considered as EMT markers, we found that they label independent cell populations in clinical specimens. Vimentin was regularly expressed both in benign epithelial glands and PCa with intact membranous E-cadherin, indicating vimentin expression does not necessarily confer mesenchymal properties to human prostate epithelial cells. Others have also demonstrated the abundant expression of vimentin in benign prostate epithelium and other epithelial cells including liver, kidney, vocal cords and breast [37-43]. Although fibronectin is an important EMT marker especially in *in vitro* studies, little is known about fibronectin expression in clinical cancers [44-49]. Expression and secretion of fibronectin allows cells to alter the extracellular matrix through the activation of matrix metalloproteinases and aid in migration [50, 51]. We showed that fibronectin is expressed in only a small percentage of tumor cells that co-expressed E-cadherin, which argues against fibronectin as a marker for EMT in clinical specimens. Taken together, the role of vimentin and fibronectin as markers of EMT is less straightforward in clinical PCa than *in vitro*.

A novel approach in our study was the investigation of molecular markers in relation to specific growth patterns instead of more general Gleason grades. Gleason grade 4 PCa encompasses a heterogeneous group of tumor growth patterns such as ill-defined, fused, glomeruloid and cribriform. While the vast majority of clinical and biological studies regard Gleason grade 4 PCa as a uniform tumor entity, we believe that discrimination of individual growth patterns adds new information to research analysis and facilitates comprehensive interpretation of research data. Recently, we and others have shown that cribriform growth in Gleason score 7 PCa is a strong independent adverse parameter for distant metastasis and disease-specific death after RP [52, 53]. Qian et al. demonstrated a copy number amplification of *c-MYC* in cribriform Gleason grade 4 PCa, but not in other Gleason grade 4 subtypes [54]. In this study, we demonstrate that N-cadherin expression preferentially occurs in ill-defined Gleason grade 4 PCa, indicating that this specific growth pattern is representing biological EMT in clinical PCa. Therefore, taking individual Gleason grade 4 growth patterns into account adds value to the interpretation of both clinical and biological PCa studies.

A potential bias in our study is the fact that our RP specimens represent a selection of intermediate risk PCa. In our institute, RP is not the first choice of therapy for a large number of patients with limited Gleason score 6 PCa on biopsy or with extensive high-risk disease. Since our population included a set of consecutive RP specimens not all growth patterns are represented equally. Although our study included the most prevalent PCa growth patterns, it cannot be excluded that the morphological substrate of EMT in pure Gleason grade 3 or less frequent Gleason grade 5 patterns is different than found in the current study. Finally, EMT refers to a dynamic process of cell transformation. Cadherin-switching is an important step in EMT, but it is unclear whether cadherin switching marks the entire or just a specific part of the dynamic EMT process.

In conclusion, we found that commonly used EMT markers vimentin, fibronectin and N-cadherin are not co-expressed within the same cell populations in clinical PCa. Since E-cadherin is downregulated in tumor cells expressing N-cadherin but not vimentin or fibronectin, cadherin switching represents the best marker of EMT in PCa patients. N-cadherin expression occurred preferentially in ill-defined Gleason grade 4 PCa, indicating that this specific growth pattern is the morphological substrate of EMT in clinical PCa.

## MATERIALS AND METHODS

### Clinical specimens

RP specimens were all retrieved from the archive of the department of pathology, Erasmus Medical Centre, Rotterdam, The Netherlands. All RP's had been performed in our institute between July 2010 and May 2013, prompted by histologically proven hormone-naïve PCa on diagnostic needle-biopsy. After receipt of RP specimens at the department of pathology, a transverse tissue slice was frozen in liquid nitrogen for research purposes. After thorough injection of 4% neutral-buffered formalin to allow for fast and equal fixation of RP specimens, the prostate was pinned to a cardboard to avoid tissue retraction. After additional fixation overnight, RP specimens were sliced transversally from apex to basis in 4 mm slices and completely embedded for pathologic analysis. At pathologic evaluation by a urogenital pathologist (GvL), modified Gleason score, extra-prostatic extension, seminal vesicle and bladder neck involvement, pT-stage (WHO 2009) and surgical margin status were all recorded. We included a primary set of 23 RP specimens and an extended set of 77 RP specimens. The primary set (*N* = 23) was used to broadly analyse the expression of EMT-associated markers vimentin, fibronectin, E- and N-cadherin, and determine whether respective markers were co-expressed in the same area. The extended set (*N* = 77) was an extension of the primary RP set (*N* = 23) with 44 novel cases and was used to statistically relate the occurrence of EMT in well-described prostate cancer growth patterns. Use of RP specimens for research purposes was approved by the Erasmus MC Medical Ethics Committee (MEC-2011-295 and 296; August 25th, 2011) and is in accordance with the Helsinki Declaration of 1975, as revised in 1983.

### Immunohistochemistry

Five μm slices were cut and mounted on aminoacetylsilane coated glass slides (Starfrost, Berlin, Germany). Slides were deparaffinized in xylene and rehydrated in decreasing ethanol steps. Endogenous peroxidase was blocked in 1% hydrogen peroxide in Phosphate Buffered Saline (PBS) for 20 min. Antigen retrieval was performed with TRIS (hydroxymethyl) aminomethane-EDTA (pH 9.0, Klinipath, Duiven, The Netherlands) in a microwave (MicroMed T/T Mega, Milestone, Sorisole, Italy) for 15 min. Slides were incubated overnight at 4°C with primary antibodies targeting N-cadherin (1:50, clone 6G11, Dako, Glostrup, Denmark), vimentin (1:500, clone Vim3B4, Dako, Glostrup, Denmark), fibronectin (1:500, ab2413, Abcam, Cambridge, U.K.) or E-cadherin (1:200, clone NCH-38, Dako, Glostrup, Denmark) diluted in phosphate-buffered normal antibody diluent (Immunologic, Duiven, The Netherlands). The specificity of all antibodies had been verified using Western blot analysis (data not shown). Secondary antibodies were incubated for 30 min. at room temperature and chromogenic visualization was performed with the EnVision Dako kit (Dako, Glostrup, Denmark). Slides were counterstained with haematoxylin, washed, dehydrated, cleared in xylene and mounted in Entellan® new (Merck Millipore, Billerica, U.S.A.). All immunohistochemical stainings included negative controls by omitting the primary antibody.

### Immunohistochemical analysis

Immunohistochemical expression analysis was performed on consecutive PCa tissue sections of a primary set of 23 RP specimens and an extended set of 77 RP's. The primary set was used to determine general expression patterns of EMT markers vimentin, fibronectin, N-cadherin and E-cadherin. In this set, expression of respective proteins was determined in benign prostate epithelium, PCa and intervening stroma. N-cadherin was additionally scored in an extended set of RP specimens (*n* = 77) to examine its expression in specific PCa growth patterns [1-3]. The occurrence and relative percentage of individual PCa growth patterns were recorded in haematoxylin and eosin stained tissue slides by a urogenital pathologist (GvL). The following growth patterns were distinguished: “glandular”, defined as well-delineated round to ellipsoid PCa glands, corresponding to Gleason grade 3; “ill-defined”, defined as PCa glands with an irregular outline; “fused”, defined as more or less complex PCa structures often with slit-like lumina with the vast majority of cells being adjacent to surround stroma; “cribriform”, defined as solid epithelial structures with punched-out lumina, with the majority of cells not contacting surrounding connective tissue; “glomeruloid”, defined as intra-glandular epithelial proliferations resembling renal glomeruli, all corresponding to Gleason grade 4; “single cells”, “cords” and “solid fields” all corresponding to Gleason grade 5 [1-3]. The percentage of N-cadherin positive tumor cells was semi-quantitatively estimated in all individual growth patterns present in one tissue section.

### Immunofluorescence

Co-expression analysis of EMT markers was performed by immunofluorescent staining of 9 fresh frozen PCa samples. Sections of 5 μm thick for were cut at a cryostat, fixed with 4% formaldehyde solution (Klinipath, Duiven, The Netherlands) for 20 min. and permeabilised with 0.5% TritonX-100 in PBS for 10 min. Slides were air-dried for one hour at room temperature, washed with PBS, and incubated with combinations of mouse anti-N-cadherin (1:25, clone 6G11, Dako, Glostrup, Denmark), rabbit anti-N-cadherin (1:75, clone D4R1H, Cell Signaling Technology, Danvers, U.S.A.), mouse anti-vimentin (1:100, clone Vim3B4, Dako, Glostrup, Denmark), rabbit anti-vimentin (1:100, clone D21H3, Cell Signaling Technology, Danvers, U.S.A.), mouse anti-E-cadherin (1:50, clone NCH-38, Dako, Glostrup, Denmark) or rabbit anti-fibronectin (1:200, ab2413, Abcam, Cambridge, U.K.) in 1% bovine serum albumin (BSA) in PBS. The specificity of all antibodies had been verified using Western blot analysis and appropriate immunohistochemical controls (data not shown). After washing with PBS, slides were incubated with goat anti-mouse Ig labelled with Alexa 488 (1:200, Molecular Probes, Eugene, U.S.A.) combined with goat anti-rabbit Ig labelled with Alexa 594 (1:200, Molecular Probes, Eugene, U.S.A.), or goat anti-rabbit Ig labelled with Alexa 488 (1:200, Molecular Probes, Eugene, U.S.A.) combined with goat anti-mouse Ig labelled with Alexa 594 (1:200, Molecular Probes, Eugene, U.S.A.). Slides were mounted in Vectashield (Vector Laboratories, Peterborough, U.K.) containing Hoechst 34580 (1:4000, Life Technologies, Bleiswijk, The Netherlands) to visualize nuclei. Immunofluorescent tissue sections were analyzed using a confocal laser scanning microscope (Zeiss LSM 700, Carl Zeiss, Oberkochen, Germany) with ZEN 2012 imaging software (Carl Zeiss, Oberkochen, Germany).

### Statistics

Comparison of EMT marker expression in benign prostate epithelium and PCa was analyzed using Pearson chi-square test. The frequency of N-cadherin expression in separate PCa growth patterns was compared using the Fisher's exact test. The non-parametric Mann-Whitney U test was used to analyze the percentage of N-cadherin positive tumor cells in individual growth patterns. A p-value of ≤ 0.05 was considered statistically significant. Statistical analyses were performed using IBM SPSS Statistics 21 (IBM Corp, Armonk, U.S.A.).
